# Mixture Proportion Design Method of Steel Fiber Reinforced Recycled Coarse Aggregate Concrete

**DOI:** 10.3390/ma12030375

**Published:** 2019-01-25

**Authors:** Danying Gao, Lijuan Zhang, Michelle Nokken, Jun Zhao

**Affiliations:** 1 School of Mechanics and Engineering Science, Zhengzhou University, No.100 Science Avenue, Zhengzhou 450001, China; gdy@zzu.edu.cn (D.G.); zhaoj@zzu.edu.cn (J.Z.); 2 Faculty of Engineering and Computer Science, Concordia University, 1455 de Maisonneuve, West, Ontreal H3G1M8, QC, Canada; m.nokken@concordia.ca

**Keywords:** recycled aggregate concrete, steel fiber, mixture proportion design, water-cement ratio, water content, sand ratio

## Abstract

Steel fiber reinforced recycled coarse aggregate concrete (SFRCAC) is an impact minimisation building material. Mixture proportion design method of SFRCAC is developed in this paper to obtain concrete with target strength and workability, which can be used in structural members. Four key parameters of mixture proportioning, steel fiber content, water-cement ratio, water content and sand ratio are discussed through the mixture design tests. The formula for calculating the four key parameters of mixture proportions for SFRCAC are established through the statistical analysis of test results, which mainly consider the influences of recycled coarse aggregate (RCA) replacement ratio and steel fiber characteristic coefficient. The detailed procedure by using the new mixture proportion design method is illustrated with examples. The formulas established have the simple form, reflect the properties of RCA and steel fibers, enhance the mixture proportion design accuracy, and provide the reference for the mix proportion design of SFRCAC.

## 1. Introduction

Recycled coarse aggregate (RCA) presents a sustainable solution to the depletion of natural coarse aggregate (NCA) resources, and plays a key role in reducing the need for landfilling the waste disposal [1,2,3,4]. Because RCA is mainly composed of two different materials, NCA and the attached cement mortar [5,6], it has much higher water absorption and lower apparent density [7,8]. As well, a large number of micro cracks are produced in the crushing process of waste concrete, which causes the higher crushing index and reduces the soundness of RCA.

Due to the poor properties, the application of RCA has been limited. Adding steel fibers to recycled coarse aggregate concrete (RCAC) can provide the bridging effect to prevent and reduce the development of inherent micro-defects in RCAC [9], improve the mechanical properties of the RCAC and especially better control its fracture process [10,11,12]. It has been well established that the incorporation of steel fibers improves the engineering performance of RCAC, including better crack resistance, increasing ductility and toughness as well as the enhancement in resistance to fatigue and impact [13]. Also, steel fiber reinforced recycled aggregate concrete has better strength and durability than recycled aggregate concrete, it can extend the service life of construction, the combination of steel fibers and RCA has great environmental and economic benefits [14]. Hence, steel fiber reinforced recycled coarse aggregate concrete (SFRCAC) has great potential for use in structural member. 

The methods for the mixture design of SFRCAC is the basis of material research as well as engineering application. The mixture proportions, such as varying coarse aggregate and steel fiber contents, will lead to the different properties of cement-based materials. Previous studies about the mixture proportion of RCAC mostly focus on the following aspects:

1. The effect of RCA on concrete strength. RCA may lead to 20–25% decrease in compressive strength compared to NCA with the same mixture proportion [15,16,17,18]. The compressive strength of RCAC depends on the RCA properties and RCA replacement ratio [19,20]. All strength grades of RCA are suitable to produce low strength concrete (20 MPa), but, the production of medium (40 MPa) and high strength (60 MPa) concrete requires the RCA to have a source strength matching or exceeding the strength of the new concrete [3].
2. The method to adjust the water content of RCAC. The water absorption and cement mortar of RCA can be reduced by surface modification or pre-soaking methods [7,21], both the pre-saturation method and the mixing water compensation method can be adopted to mix RCAC [22]. For the latter, a two-stage mixing approach divides the mixing process into two parts and proportionally splits the required water into two, this method has been found to improve the strength of RCAC [23,24]. However, the methods mentioned above only make some adjustments of water content in the batching process and do not provide the suitable calculation method for water content of RCAC.
3. The equivalent mortar volume method [25]. This method considers RCA as a two-phase material including mortar and NCA. The mixture design principle is the quantity and quality of each phase of RCAC should achieve the same total mortar volume with NCAC, but the calculation process of the method is too complicated to be easily used by engineers. A new mixture design method was put forward by taking into account the higher porosity of RCAs, but now it’s only in conceptual and the RCA replacement ratio can’t reach 100% [26].

Although ACI-555R [27] provides the guidelines for proportioning of concrete mixtures made with RCA, neither it nor any other source gives a specific mixture design method for achieving target fresh and hardened properties for SFRCAC. Therefore, to obtain a specific and effective mixture design method for SFRCAC to ensure it has the similar fresh and hardened properties as conventional NCAC even if using the different RAC replacement ratios is the main aim of this study. The calculating formulas of steel fiber content, water-cement ratio, water content and sand ratio are proposed by extensive experiments and analysis.

## 2. Materials and Methods 

### 2.1. Materials

Portland cement (P.O 42.5) conforming to the stipulation in GB 175 [28] was used in all mixtures. The coarse aggregate included NCA and RCA. NCA was crushed limestone with continuous grading of particle sizes from 4.75 mm to 20 mm. RCA was obtained from the waste commercial ready-mixed concrete in a concrete testing station with the compressive strength ranging from 30 MPa to 50 MPa, and unknown age. The waste concrete was collected and crushed through a jaw crusher, then screened through sieves with a maximum size of 20 mm and a minimum size of 4.75 mm. The grading of the NCA and RCA was within the upper and lower limit bounds of ASTM C33 [29] except that the particle size distribution was in the percentage passing the 9.5 mm sieve, and 24% for NCA, 35% for RCA. The fine aggregate was river sand with a fineness modulus of 2.67. The properties of all aggregates used in the test are shown in Table 1. The test was carried out according to Chinese standard GB/T 25177 [30] and GB/T 14685 [31]. The test method of crush index is pressuring the coarse aggregate with the size from 9.5 mm to 19 mm which is put into the specified modulus to 200 kN with the speed of 1 kN/s, and then the mass of these coarse aggregate which size is below 2.36 mm is measured and the crush index was calculated. Crush index mainly reflects the soundness of coarse aggregate. It can be seen from Table 1 that the crush index of RCA is much higher than NCA, it means that the soundness of RCA is poorer than NCA. The void ratio of coarse aggregate is calculated by the difference between their apparent density and bulk density. The void ratio mainly reflecting the compactness of coarse aggregate, the higher of the void ratio, the poorer of compactness of coarse aggregate. It can be seen from Table 1 that the void ratio of RCA is higher than NCA.

Compared with the NCA, the RCA had higher water absorption, void ratio and crushing index, but lower apparent density. All aggregates used were in the oven-dry condition. Three types of steel fiber were used in this study, the photos and characteristics of steel fibers are shown in Table 2.

### 2.2. Mixture Proportion Method

The research objective of this paper is to precisely determine the mixture design parameters of SFRCAC to achieve the target strength and workability. Generally, the conventional concrete is mainly composed of natural coarse aggregate, sand, cement and water. The key mixture design parameters for concrete include water-cement ratio (W/C), water content (*m*_w_) and sand ratio (*β*_s_). Once these parameters are determined, each component can be calculated by forecast quality method or absolute volume method [32]. 

Compared with conventional concrete, the properties and amounts of RCA and steel fiber will inevitably affect the mechanical properties and workability of SFRCAC. Therefore, how to determine the effect of RCA and steel fiber on the mixture design parameters is the first problem to be solved. The experiments in this paper was divided into four parts and the test parameters used in the test are listed in Table 3.

According to the test purpose, the whole test was divided into the following four parts:

1. The purpose of the first part was the determination of the steel fiber content. Steel fibers have more significant effect on the flexural strength than compressive strength [33], hence, the volume fraction of steel fiber (*V*_f_) can be determined by the flexural strength achieved. In order to ensure the workability of SFRCAC, for each increase in *V*_f_ of 0.5%, the water content is increased by 8 kg/m^3^ and the sand ratio by 3% [34]. To accurately determine the reinforcement effect of steel fibers on flexural strength, one mixture of the plain concrete without steel fibers made with the same mixture proportion corresponding to each *V*_f_ group of SFRCAC was also prepared. The mixture designs used in this part are listed in Table 4.
2. The water-cement ratio (W/C) is the parameter influence concrete strength in mixture design. Previous research has shown that using the same W/C, the addition of the steel fiber does significantly improve the compressive strength of concrete after 28 days [35], but RCA may lead to 20–25% decreasing of compressive strength [15]. The properties and replacement ratio of RCA have significant effect on the strength of SFRCAC. The relation between compressive strength (*f*_cu_) and W/C was studied in this part; RCA replacement ratio (*r*_g_), which is the mass ratio of the RCA to the total coarse aggregate, was taken as 0%, 50% and 100%. Due to the differences in density of NCA and RCA, the quantity of aggregate and sand was increasing with the increase of W/C in the mixing process. The mixture designs used in this part are listed in Table 5.
3. Slump is an index reflecting the workability of concrete, which mainly was affected by the water content of concrete. The accurate determination for the water content of SFRCAC with different RCA and steel fibers to achieve target slump is the objective of this part. More water is required in the batching process to obtain the similar slump for RCAC as NCAC due to the higher water absorption of RCA [21,36,37]. The slump of concrete has been found to decrease with the increase of aspect ratio and volume fraction of steel fiber [38]. In this part, the several mixtures were made at each water content varying both *r*_g_ and *V*_f_, as shown in Table 6.
4. The sand ratio (*β*_s_) is the mass ratio of sand to the total mass of aggregates (the mass sum of sand and coarse aggregate). Generally, *β*_s_ is chosen according to the experience for ordinary concrete [33]. Now, there is no precise calculation formula of *β*_s_ for SFRCAC. The determination of a reasonable sand ratio for SFRCAC was studied in this part. According to the principle that fine aggregate needs to fill the voids between coarse aggregates, the volume of sand required in SFRCAC should be the sum of voids caused by all coarse aggregates (including NCA and RCA) and the dispersal of steel fibers. Hence, a new calculation model of sand volume can be set up as follows:
  (1) $$V_{s}=\gamma \times \left(V_{\mathrm{na}}\times P_{\mathrm{na}}+V_{\mathrm{ra}}\times P_{\mathrm{ra}}+V_{f}\right)$$
  where, *γ* is the sand rich coefficient, it is the volume ratio between the fine aggregate and the void caused by coarse aggregate and steels fiber, the range of *γ* can be taken from 1.1 to 1.4 for NCAC [39], and can also be determined by tests; *P*_na_ is the void ratio of NCA, *P*_ra_ is the void ratio of RCA, both *P*_na_ and *P*_ra_ are the basic material properties of coarse aggregate and can be obtained by tests; *V*_f_ is the volume fraction of steel fibers used to represent the voids caused by steel fibers, because the void caused by steel fibers should be less than the volume of steel fibers in the common use range of 0–2% and rarely more than 4%. The sand content can be calculated by Equation (1), the values of other mixture design parameters in this part are consistent with the previous parts. Table 7 gives the mixture designs in this part.

When the material and mixture design parameters were determined, the “absolute volume method” was chosen to calculate the components of SFRCAC because the changing range of RCA density is bigger than NCA, which leads to the weight per cubic meter of SFRCAC is difficult to estimate. The dosage of each component of SFRCAC can be calculated by:

(2) $$V_{c}+V_{w}+V_{s}+V_{\mathrm{na}}+V_{\mathrm{ra}}+V_{f}+\alpha =1$$

(3) $$r_{g}=\frac{m_{\mathrm{ra}}}{m_{\mathrm{ra}}+m_{\mathrm{na}}}=\frac{\rho_{\mathrm{ra}}\times V_{\mathrm{ra}}}{\rho_{\mathrm{ra}}\times V_{\mathrm{ra}}+\rho_{\mathrm{na}}\times V_{\mathrm{na}}}\Rightarrow \frac{V_{\mathrm{na}}}{V_{\mathrm{ra}}}=\frac{(1-r_{g})\times \rho_{\mathrm{ra}}}{r_{g}\times \rho_{\mathrm{na}}}$$

(4) $$\beta_{s}=\frac{m_{s}}{m_{s}+m_{\mathrm{na}}+m_{\mathrm{ra}}}=\frac{\rho_{s}V_{s}}{\rho_{s}V_{s}+\rho_{\mathrm{na}}V_{\mathrm{na}}+\rho_{\mathrm{ra}}V_{\mathrm{ra}}}$$

where, *V*_c_, *V*_w_, *V*_s_, *V*_na_, *V*_ra_ and *V*_f_ is the volume of cement, water, sand, NCA, RCA and steel fibers, respectively; *m*_s_, *m*_na_ and *m*_ra_ are the mass of sand, NCA and RCA, respectively; *ρ*_na_ and *ρ*_ra_ are the apparent density of sand, NCA and RCA, respectively; α is the air content, to non-air-entrained steel fiber concrete, α = 0.02.

### 2.3. Specimen Preparation and Test

The cubic specimens with side length of 150 mm were cast for the compressive strength (*f*_cu_) test, the prism specimens of 100 mm × 100 mm × 400 mm were cast for flexural strength (*f*_ftm_) test, in which the three specimens for each group were prepared. The mixing process included three steps. Firstly, the suitable moulds were prepared and brushed inside with a release agent. Secondly, all aggregates and steel fibers were put into a small mixer to mix for about 2 min to ensure that steel fibers could be uniformly distributed in the aggregates. Thirdly, the cement was added, and mixing continued for another minute. Finally, the water was added to the mixer slowly, and mixed for another 2 min.

The slump of fresh SFRCAC was tested first, then was put into the prepared moulds and vibrated for 20 s. After 24 h curing in ambient temperature, the specimens were carefully demoulded and placed in a curing room at approximately 95% RH and 20 °C.

All the tests were conducted after the 28-days curing of specimens. The compressive tests were performed according to the stipulation in GB/T50081 [40], and were carried on a servo-hydraulic closed-loop testing machine with capacity of 3000 kN at the loading rate of 0.6 MPa/s. The flexural tests were carried on a MTS810 testing machine with capacity of 500 kN, displacement control at a rate of 0.1 mm/min, according to ASTM C1609 (Using Beam With Third-Point Loading) [41]. The test results of each group are the mean value of test results for three specimens. The test results in this research are listed in Table 4, Table 5, Table 6 and Table 7, respectively.

## 3. Analysis and Discussion

### 3.1. Steel Fiber Content

Based on the experimental results in Table 4, the flexural strength increases as *V*_f_ increases from 0 to 2%, regardless if *r*_g_ = 0 or *r*_g_ = 100%. This indicates that the higher the *V*_f_, the better the reinforcing effect of steel fiber on flexural strength. Because the fibers of MF and WF have similar aspect ratios, the effect of MF and WF on the flexural strength of RCAC and NCAC is quite similar. The aspect ratio of HF is much higher than MF and WF, and consequently the flexural strength and reinforcement ratio of HF is much higher.

**Figure 1:**
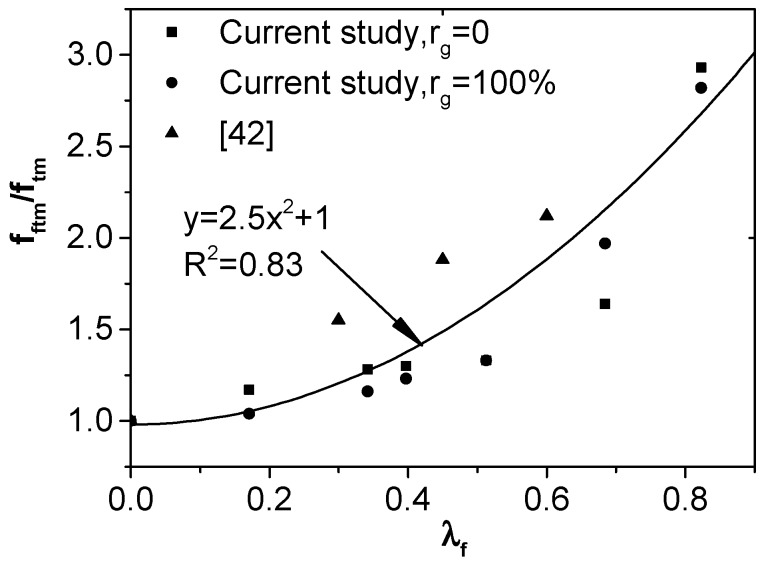
Relation between *f*_ftm_/*f*_tm_ and *λ*_f_.

According to the analysis mentioned above, the volume fraction (*V*_f_) and aspect ratio (*l*_f_/*d*_f_) of steel fibers have much influence on the flexural strength of RCAC, which can be comprehensively reflected by steel fiber characteristic coefficient (*λ*_f_), where *λ*_f_ = *V*_f_*l*_f_/*d*_f_. The relation of the flexural strength ratio of SFRCAC to RCAC with steel fiber characteristic coefficient, based on the experimental data from this paper and previous literature [42], is shown in Figure 1, an equation is put forward as follows:

(5) $$f_{\mathrm{ftm}}/f_{\mathrm{tm}}=\alpha_{f}\lambda_{f}^{2}+\beta_{f}$$

where, *f*_ftm_ is the flexural strength of SFRCAC, MPa; *f*_tm_ is the flexural strength of RCAC which has the same mix proportion corresponding to SFRCAC, MPa; *α*_f_ and *β*_f_ are the parameters related to material properties, here, *α*_f_ =2.5, *β*_f_ =1.

The steel fiber characteristic coefficient can be calculated by Equation (5) for the required flexural strength of SFRCAC, then the steel fiber volume fraction can be determined after choosing the appropriate steel fiber type and aspect ratio.

### 3.2. Water-Cement Ratio

The compressive strength test results of RCAC with different W/C are listed in Table 5. Obviously, the compressive strength *f*_cu_ of RCAC decreases with the increase of *r*_g_ at the same W/C because the quality of the RCA is lower than NCA as stated in many previous studies [6,15,43,44].

For the RCAC with *r*_g_ of 50% or 100% and W/C of above 0.35, *f*_cu_ increases continuously with the decrease of W/C, but for the RCAC with W/C of below 0.35, *f*_cu_ decreases slightly with the decrease of W/C. This is different from the NCAC with *r*_g_ of 0%, where *f*_cu_ decreases continuously with the increase of W/C. This is because that the failure of RCAC is caused by the damage of weak point inherited from crushed RCA rather than the damage of cement mortar when W/C is below 0.35 and compressive strength is higher than 50 MPa. Therefore, the compressive strength of RCAC is mainly limited by the strength of RCA instead of W/C. Previous studies have had the similar conclusions [3,16]. Consequently, the weakest point in concretes made with RCA of medium–high strength (45–60 MPa) could be determined by the strength of RCA or the adhered mortar [3,16].

Based on the stipulation in Chinese Standards JGJ55 [32], the first step in mixture design is to determine the W/C according to the target compressive strength by using the following equation:

(6) $$f_{\mathrm{cu},0}=\alpha_{a}f_{\mathrm{ce}}(C/W-\alpha_{b})$$

where *f*_cu,0_ is the cubic compressive strength of concrete at 28 days, MPa; *f*_ce_ is the compressive strength of cement at 28 days, MPa; W/C is the cement-water ratio; *α*_a_ is the cement strength conversion coefficient; *α*_b_ is the virtual cement-water ratio. *α*_a_ and *α*_b_ are dependent on the quality and type of coarse aggregate. When the coarse aggregate is gravel, *α*_a_ = 0.53, *α*_b_ = 0.2; when the coarse aggregate is pebble, *α*_a_ = 0.49, *α*_b_ = 0.13.

**Figure 2:**
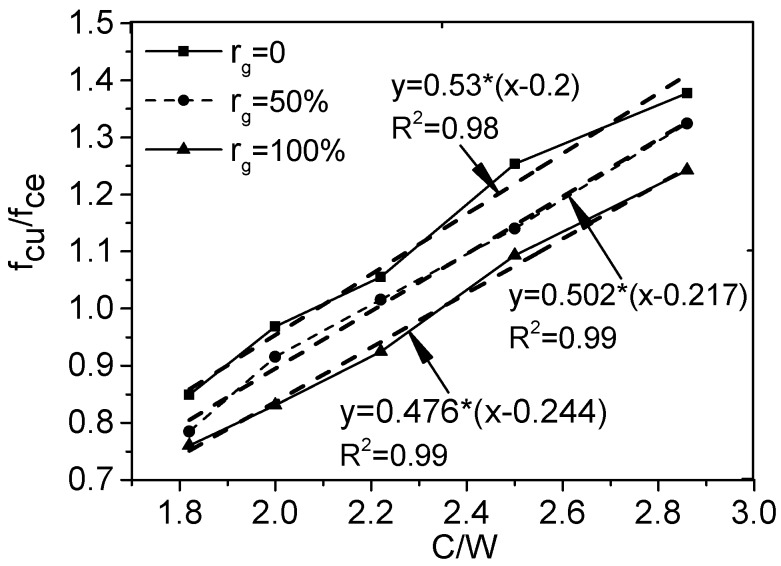
Relation between *f*_cu_/*f*_ce_ and C/W.

When W/C changes from 0.35 to 0.55, the relation between the ratio of RCAC compressive strength to cement compressive strength *f*_cu_/*f*_ce_ and C/W is shown in Figure 2, in which the solid points stand for the test results. It is clear that *f*_cu_/*f*_ce_ is proportional to C/W for the RCAC with the constant of *r*_g_, which abides by the principle of Equation (6). The values of *α*_a_ and *α*_b_ for SFRCAC with different *r*_g_ can be obtained through the regression analysis of the experimental data, that is *α*_a_ = 0.53 and *α*_b_ = 0.2 for *r*_g_ = 0%; *α*_a_ = 0.502 and *α*_b_ = 0.217 for *r*_g_ = 50%; *α*_a_ = 0.476 and *α*_b_ = 0.244 for *r*_g_ = 100%.

The quality of the coarse aggregate used in RCAC is not only related to the property of RCA, but also *r*_g_. Generally, the quality of the RCA is weaker than NCA, *α*_a_ decreases and *α*_b_ increases with the increase of *r*_g_, respectively. Therefore, the relation of *α*_a_, *α*_b_ with *r*_g_ can be expressed as follows:

(7) $$\begin{array}{l} \alpha_{a}=0.53(1-\alpha_{c}\times r_{g})\\ \alpha_{b}=0.2(1+\alpha_{d}\times r_{g}) \end{array}$$

where, *α*_c_ and *α*_d_ are the coefficient related to the quality of recycled aggregate. Putting the values of *α*_a_ and *α*_b_ gotten from test results into the Equation (7), the value of *α*_c_ and *α*_d_ for the RCA has been determined to be 0.1 and 0.2, resulting in the following equations:

(8) $$\begin{array}{l} \alpha_{a}=0.53(1-0.1\times r_{g})\\ \alpha_{b}=0.2(1+0.2\times r_{g}) \end{array}$$

When RCA belongs to category II and the target compressive strength is below 50 MPa, Equation (8) can be used to determine *α*_a_ and *α*_b_ for RCAC. For other kinds of RCA, the value of *α*_c_ and *α*_d_ may be different, and the suitable value must be determined through the regression analysis of experimental data by Equation (7), then put *α*_a_ and *α*_b_ into Equation (6) to calculate the water–cement ratio.

### 3.3. Water Content

#### 3.3.1. Influence of Recycled Coarse Aggregate

**Figure 3:**
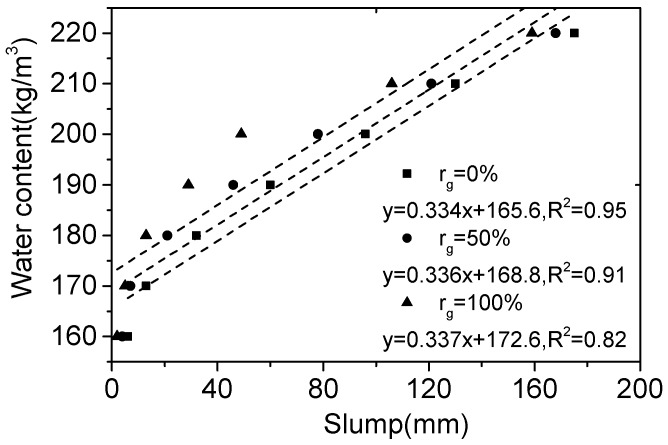
Relation between water content and slump of RCAC.

According to the experimental results in Table 6, the relation between the water content and slump of RCAC is shown in Figure 3. It can be seen that when *r*_g_ is kept constant, the slump of RCAC increases with the rise of water content. With the same water content, the slump of RCAC reduces with the increase of *r*_g_. This is because RCA has higher water absorption than NCA, and absorbs more water in concrete batching, which leads the reduction of effective water and the decreasing of slump.

Generally, the water content is determined by the maximum size of coarse aggregate and the desired range of slump. In China, the water content of ordinary concrete is generally estimated through table look-up or calculated with equation as follows [39]:

(9) $$m_{w}=3.33\times \left(0.1\times T+K\right)$$

where *m*_w_ is the water content, kg/m^3^; *T* is the desired slump, mm; *K* is the constant determined by the type and maximum size of coarse aggregate. With the different type of aggregate, the value of *K* can be obtained by test or look-up table, *K* = 53 for the coarse aggregate of rubble with the maximum size of 20 mm, and *K* = 48.5 for the coarse aggregate of rubble with the maximum size of 40 mm. It can be seen that the larger the particle diameter, the smaller the *K* value.

Based on the test data in this paper, the formulas for water content can be obtained by regression and represented in Figure 3. It indicates the relation of water content with slump is a linear function for the SFRCAC with different *r*_g_, the slopes of these linear functions are similar, but the constant terms increase with the increasing of *r*_g_. Therefore, the relation between water content and slump can be expressed as follows:

(10) $$m_{w}=3.33\times \left(0.1\times T_{0}+K_{g}\right)$$

where *T*_0_ is the slump of RCAC; *K*_g_ is the parameter relative to the type and maximum size of coarse aggregate. According to the regression analysis of test data in this paper, *K*_g_ is obtained as 49.7, 50.7 and 51.8 for *r*_g_ of 0, 50% and 100%, respectively. *K*_g_ is no longer a constant like *K* in Equation (9), but it increases with the increasing of *r*_g_. This is consistent with that the water content of RCAC increases with the increasing of *r*_g_. The difference of water absorption between RCA and NCA is an important factor affecting water content of RCAC. Hence, the calculation formula of *K*_g_ can be expressed as follows:

(11) $$K_{g}=K[1+(\omega_{\mathrm{ra}}-\omega_{\mathrm{na}})\times r_{g}]$$

where *ω*_ra_ is the water absorption of RCA; *ω*_na_ is the water absorption of NCA; *K* is the constant depending on the type and maximum size of NCA, its value can be obtained by test or look-up table.

In this study, the value of *K* is 49.7 from the test results, and *ω*_na_ = 1.4%, *ω*_ra_ = 4.85%, as shown in Table 1. Putting the value of these parameters into Equation (11), getting *K*_g_ = 50.6 for *r*_g_ = 50% and *K*_g_ = 51.4 for *r*_g_ = 100%. The value of *K*_g_ with the different *r*_g_ calculated by Equation (11) was very close to the test results. Therefore, the water content of RCAC can be determined by Equations (10) and (11).

#### 3.3.2. Influence of Steel Fibers

Based on the test results, the relation between the slump of SFRCAC and *V*_f_ can be seen in Table 7. Obviously, the slump decreases with the increasing of *V*_f_. For each type of steel fiber, the slump of SFRCAC decreases with the increasing of *r*_g_. Due to the larger aspect ratio of HF, the slump reduction of RCAC with HF is much higher than that of MF, and has little to do with *r*_g_. Besides, the similar slump reduction was observed irrespective of the aggregate type.

**Figure 4:**
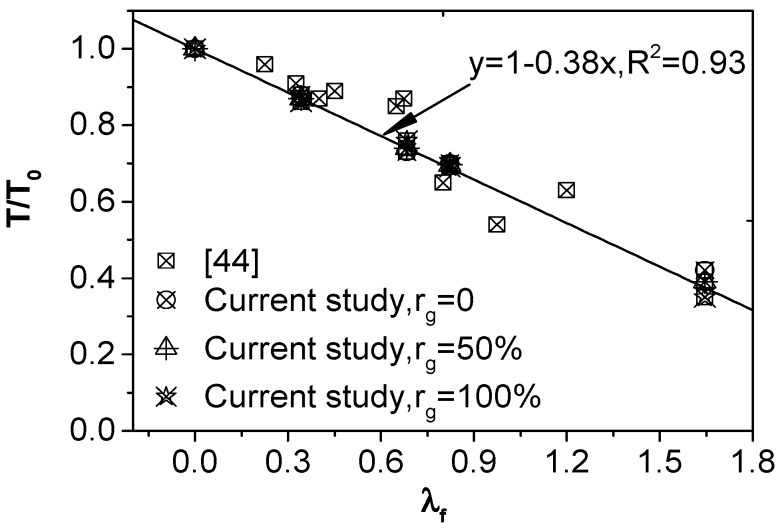
Relation between *T*/*T*_0_ and *λ*_f_.

The relation between the slump reduction ratio and the characteristic coefficient of steel fiber (*λ*_f_) is shown in Figure 4, in which the points are drawn from the test results in this paper and previous literature [44]. Obviously, the slump reduction ratio is approximately linear with the characteristic coefficient of steel fiber, a formula can be determined based on the test results:

(12) $$T/T_{0}=1-0.38\lambda_{f}$$

where *T* is the desired slump of SFRCAC, mm; *T*_0_ is the slump of the plain concrete which is corresponding to SFRCAC in same mixture proportion, mm. When the desired slump of SFRCAC (*T*) is determined, *T*_0_ can be calculated by Equation (12), then using Equation (10), the water content of SFRCAC can be determined.

### 3.4. Sand Ratio

The test results by using the new sand content method determined by Equation (1) are shown in Table 7. It can be seen that sand ratio *β*_s_ increases regularly with the increasing of *V*_f_ and *r*_g_. For the SFRCAC with the same *V*_f_, *β*_s_ increases by 2.2% as *r*_g_ increases by 50%. For the SFRCAC with the same *r*_g_, *β*_s_ increases by 0.7% as *V*_f_ increases by 0.5%. This result is very close to that for the SFNCAC [45], where steel fiber was treated as part of the coarse aggregate used in sand ratio calculation, and it was concluded that *β*_s_ increases by 0.8% as *V*_f_ increases by 0.5%.

**Figure 5:**
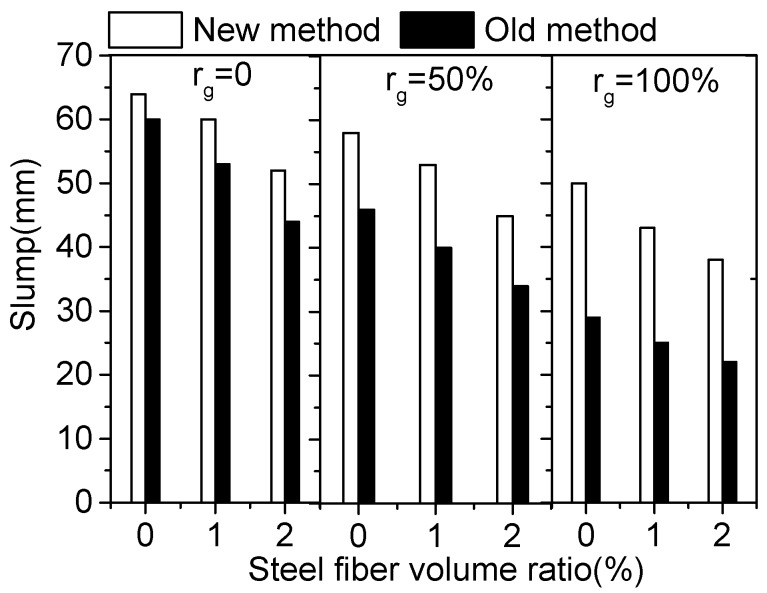
Comparison of slump by using different sand ratio method.

In the test of this paper, the water content is kept constant, and the slump still decreases obviously with the increasing of *V*_f_ and *r*_g_ although the sand ratio increases with the increasing of *V*_f_ and *r*_g_. However, the slump in Table 7 using the new sand ratio method is much higher than that in Table 6, where the sand ratio was a constant of 36%. The comparison of these test results is shown in Figure 5. Obviously, the slump of SFRCAC using the new sand ratio method is much higher than that by using the old method, especially for SFRCAC with *r*_g_ of 100%. This indicates that the workability of SFRCAC can be improved by using the new sand ratio method.

**Figure 6:**
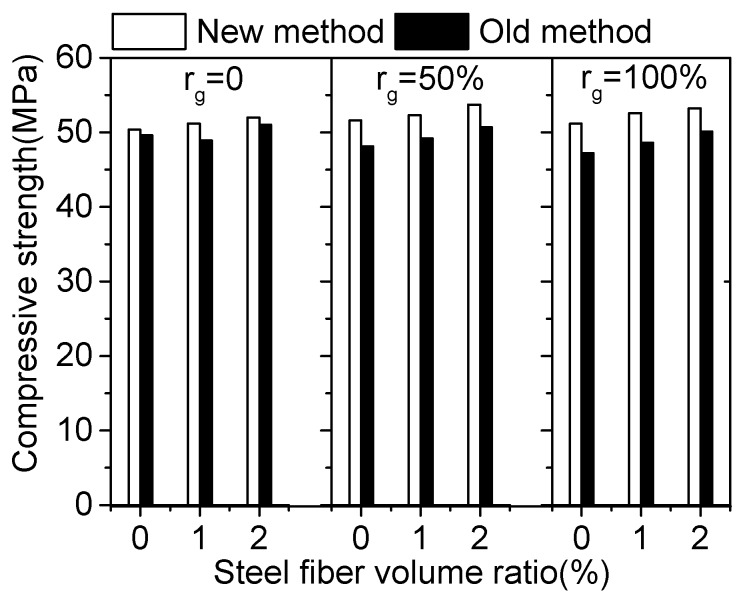
Comparison of compressive strength by using different sand ratio method.

The comparison of measured compressive strength of SFRCAC designed by the new and old sand ratio method is shown in Figure 6. It can be seen from Figure 6 that for the SFRCAC with a constant of *r*_g_, its compressive strength increases with the increasing of *V*_f_. When *r*_g_ increases from 0 to 100%, the compressive strength of SFRCAC designed by using the old sand ratio method decreases, but that designed by using the new sand ratio method increases slightly. For SFRCAC with *r*_g_ of 100%, the difference of the compressive strength of SFRCAC designed by the new and old sand ratio method is more remarkable.

## 4. Experimental Verification

In order to verify the mixture proportion design method was put forward in this paper, the specimens with the different dosages of RCA (*r*_g_ = 0%, 50%, 100%) were used to achieve a desired compressive strength (*f*_cu_ = 40 MPa), flexural strength (*f*_ftm_ =10 MPa), and slump (*T* = 50 mm). The design process is shown as follows.

1. Determination of the steel fiber content
  Checking whether steel fiber is necessary or not. An empirical formula between the flexural strength and compressive strength for normal concrete [46] is used here, *f*_tm_ = 0.89 × ($f_{c}^{\prime }$)^0.5^, where $f_{c}^{\prime }$ is cylinder compressive strength and $f_{c}^{\prime }=0.8f_{\mathrm{cu}}$.
  The predicted value of *f*_tm_ = 0.89 × (0.8 × 40)^0.5^ = 5.03 MPa; since, *f*_tm_ < 10 MPa (the desired flexural strength), it is necessary to add the steel fiber to obtain the desired flexural strength.
2. Calculating the required content of steel fibers [Equation (5)]
  $\lambda_{f}=\sqrt{(f_{\mathrm{ftm}}/f_{\mathrm{tm}}-\beta_{f})/\alpha_{f}}=\sqrt{(10/5.03-1)/2.51}=0.63$
  Since *λ*_f_ = *V*_f_*l*_f_/*d*_f,_ *V*_f_ = 0.63/34.2 = 1.84% for MF; *V*_f_ = 0.63/39.7 = 1.6% for WF; *V*_f_ = 0.63/82.3 = 0.76% for HF.
3. Calculating the required W/C [Equations (6) and (8)]
  According to Equation (8), *α*_a_ = 0.53 × (1 − 0.1 × *r*_g_) and *α*_b_ = 0.2 × (1 + 0.2 × *r*_g_);
  *α*_a_ = 0.53, *α*_b_ = 0.2 for *r*_g_ = 0; *α*_a_ = 0.503, *α*_b_ = 0.22 for *r*_g_ = 50%; *α*_a_ = 0.477, *α*_b_ = 0.24 for *r*_g_ = 100%.
  *f*_cu,0_ is the prepared strength, σ is the standard deviation of compressive strength. According to Chinese Standard [47], for the RCAC with *f*_cu_ = 40 MPa, σ = 6 MPa, *f*_cu,0_ = 40 + 1.645 × 6 = 50 MPa.
  According to Equation (6), *f*_cu,__0_/*f*_ce_ = *α*_a_ × (C/W − *α*_b_), C/W = 1/(*f*_cu,__0_/*f*_ce_/*α*_a_ + *α*_b_), from Table 1, *f*_ce_ = 45 MPa, then: W/C = 1/(50/45/0.53 + 0.2) = 0.43, for *r*_g_ = 0; W/C = 1/(50/45/0.503 + 0.22) = 0.41, for *r*_g_ = 50%; W/C = 1/(50/45/0.477 + 0.24) = 0.39, for *r*_g_ = 100%.
4. Calculating the required water content [Equations (10)–(12)]
  According to Equation (11), *K*_g_ = *K* × [1 + (*ω*_ra_ − *ω*_na_) × *r*_g_], *K* = 49.7. From Table 1, *ω*_na_ = 1.4%, *ω*_ra_ = 4.85%. *K*_g_ = 49.7 × [1 + (0.0485 − 0.014) × *r*_g_], *K*_g_ = 49.7 for *r*_g_ = 0; *K*_g_ = 50.6 for *r*_g_ = 0; *K*_g_ = 51.4 for *r*_g_ = 100%.
  According to Equation (12), *T*/*T*_0_ = 1 − 0.38*λ*_f_, *T*_0_ = *T*/(1 − 0.38*λ*_f_) = 50/(1 − 0.38 × 0.63) = 66 mm.
  According to Equation (10), *m*_w_ = 3.33 × (0.1*T* + *K*_g_), then
  *m*_w_ = 3.33 × (0.1 × 66 + 49.7) = 187.5 kg, for *r*_g_ = 0; *m*_w_ = 3.33 × (0.1 × 66 + 50.6) = 190.5 kg, for *r*_g_ = 50%; *m*_w_ = 3.33 × (0.1 × 66 + 51.4) = 193 kg, for *r*_g_ = 100%.
5. Calculating the required aggregate dosage [Equations (1)–(3)].
  *r*_g_ = 50% and WF was taken as an example to explain the calculation.
  From Table 1, *P*_na_ = 44.3%, *P*_ra_ = 50.3%, *ρ*_na_ = 2814 kg/m^3^, *ρ*_ra_ = 2640 kg/m^3^; when *r*_g_ = 50%, *m*_na_ = *m*_ra_, *V*_na_ = 0.938 × *V*_ra_;
  According to Step 1, *V*_f_ = 1.6% for WF; and then put these parameters in Equation (1),
  *V*_f_ = 1.4 × (0.443 × 0.938 × *V*_ra_ + 0.503 × *V*_ra_ + 0.016) = 1.286 × *V*_ra_ + 0.0224.
  According to Steps 2 and 3, *r*_g_ = 50%, W/C = 0.41, *m*_w_ = 190.5 kg; therefore, *m*_c_ = *m*_w_/(W/C) = 464.6 kg; *V*_w_ = *m*_w_/*ρ*_w_ = 0.1905 m^3^, *V*_c_ = *m*_c_/*ρ*_c_ = 464.5/3100 = 0.15 m^3^.
  According to Equation (2), *V*_c_ + *V*_w_ + *V*_s_ + *V*_na_ + *V*_ra_ + *V*_f_ + α = 1; 0.15 + 0.1905 + (1.286 × *V*_ra_ + 0.0224) + 0.938 × *V*_ra_ + *V*_ra_ + 0.016 + 0.02 = 1, *V*_ra_ = 0.187, *m*_ra_ = *V*_ra_ × *ρ*_ra_ = 493.7 kg; *V*_s_ = 1.286 × *V*_ra_ + 0.0224 = 0.263, *m*_s_ = *V*_s_ × *ρ*_s_ = 672.3 kg.

Based on the established method, shown as above example, the mixture proportion details of SFRCAC are represented in Table 8, and the relevant test results of compressive strength, flexural strength and slump of SFRCAC are shown in the identical table. It can be seen that all groups of SFRCACs have achieved the target compressive strength, flexural strength and slump.

## 5. Conclusions

Through the above analysis, the mixture design method of SFRCAC which is given the target compressive strength, flexural strength and slump is following:

1. The flexural strength of RCAC can be improved by adding steel fibers. The characteristic coefficient of steel fiber can be calculated by Equation (5). Once the steel fiber type is chosen, the steel fiber volume fraction can be determined.
2. The calculation model of water–cement ratio for normal concrete can also be used in recycled concrete, except that the parameter *α*_a_, *α*_b_, which are connected with the quality of coarse aggregate and the replacement ratio (*r*_g_), should be determined by test or calculated by Equations (6) and (7).
3. The water content of SFRCAC is related to replacement ratio (*r*_g_) and characteristic coefficient of steel fiber (λ_f_). According to the desired slump, the slump can be calculated by Equation (12). Then, the water content can be calculated by Equations (10) and (11).
4. The sand ratio (*β*_s_) of SFRCAC is related to void fraction and apparent density of coarse aggregate, replacement ratio (*r*_g_) and characteristic coefficient of steel fiber (*λ*_f_). The volume fraction of steel fiber (*V*_s_) can be calculated by Equation (1). Based on the material property of aggregates and steel fiber used in the test, *β*_s_ increases by a factor 2.4% when *r*_g_ is increased by 50%, *β*_s_ increases by a factor 0.7% when *V*_f_ is increased by 0.5%.
5. Due to the apparent density of RCA being generally less than that of NCA, the mass of RCAC is greatly influenced by apparent density and replacement ratio (*r*_g_), the absolute volume method is recommended to be used to calculate the material component of the SFRCAC.

## Figures and Tables

**Table 1:** Physical properties of the coarse and fine aggregate.

Aggregate Type	Apparent Density (kg/m^3^)	Loose Packing Density (kg/m^3^)	Dry-Rodded Density (kg/m^3^)	Water Absorption (wt. %)	Crush Index (%)	Void Ratio (%)
RCA	2640	1302	1412	4.85	17.7	50.3
NCA	2814	1568	1630	1.40	8.8	44.3
Sand	2556	1611	1486	0.56	–	–

**Table 2:** Photos and characteristics of steel fibers.

**Steel Fiber Type**	**MF**	**WF**	**HF**
**Fiber photo**	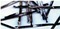	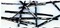	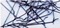
**Mean length (l_f_)/mm**	32.3	30	62
**Mean diameter (d_f_)/mm**	0.94	0.76	0.75
**Aspect ratio (l_f_/d_f_)**	34.2	39.7	82.3

**Table 3:** Test plan and parameters.

Test Parameters	Material Parameters			Mixture Design Parameters			Assessment Criteria
	Steel Fiber Type	*V*_f_	*r*_g_	W/C	*m*_w_	*β*_s_	
Steel fiber content	MF/WF/HF	0–2%	0, 100%	0.48	164–196	36%	*f*_ftm_
Water-cement ratio	none	–	0, 50%, 100%	0.30–0.55	180	36%	*f*_cu_
Water content	MF/HF	0–2%	0, 50%, 100%	0.40	160–220	36%	slump
Sand content	MF	0–2%	0, 50%, 100%	0.40	190	New method	*f*_cu_, slump

**Table 4:** Mixture design (kg/m^3^) and flexural strength of steel fiber reinforced recycled coarse aggregate concrete (SFRCAC) with different steel fiber.

Specimen No.	Water	Cement	Sand	NCA	RCA	Steel Fiber	*f*_ftm_/MPa
R0	164	342	721	1283	0	0	5.90
R100	164	342	721	0	1283	0	4.46
R0MF0.5	172	358	749	1171	0	39	6.67
R0NF0.5-C	172	358	749	1171	0	0	5.71
R100MF0.5	172	358	749	0	1171	39	5.00
R100NF0.5-C	172	358	749	0	1171	0	4.79
R0MF1.0	180	375	796	1099	0	78	7.28
R0HF1.0	180	375	796	1099	0	78	16.25
R0WF1.0	180	375	796	1099	0	78	8.13
R0NF1.0-C	180	375	796	1099	0	0	5.78
R100MF1.0	180	375	796	0	1099	78	5.44
R100HF1.0	180	375	796	0	1099	78	14.07
R100WF1.0	180	375	796	0	1099	78	5.73
R100NF1.0-C	180	375	796	0	1099	0	4.79
R0MF1.5	188	392	842	1029	0	117	8.76
R0NF1.5-C	188	392	842	1029	0	0	6.59
R100MF1.5	188	392	842	0	1029	117	7.36
R100NF1.5-C	188	392	842	0	1029	0	5.53
R0MF2.0	196	408	886	960	0	156	9.27
R0NF2.0-C	196	408	886	960	0	0	5.65
R100MF 2.0	196	408	886	0	960	156	8.54
R100NF2.0-C	196	408	886	0	960	0	4.34

Note: R100MF1.0 stands for the specimen with *r*_g_ of 100%, milling steel fiber and *V*_f_ of 1.0%; R100NF1.0-C is the plain concrete which has the same mix proportion with R100F1.0.

**Table 5:** Mixture design (kg/m^3^) and compressive strength of recycled coarse aggregate concrete (RCAC) with different water-cement ratio (W/C).

Specimen No.	Water	Cement	Sand	NCA	RCA	*f*_cu_/MPa
W/C0.3R0	180	600	603	1073	0	75.2
W/C 0.3R50	180	600	590	525	525	58.4
W/C0.3R100	180	600	579	0	1030	55.3
W/C0.35R0	180	514	630	1121	0	62.0
W/C0.35R50	180	514	617	549	549	59.6
W/C0.35R100	180	514	605	0	1076	55.9
W/C0.4R0	180	450	650	1157	0	56.4
W/C0.4R50	180	450	636	566	566	51.3
W/C0.4R100	180	450	624	0	1111	49.2
W/C0.45R0	180	400	666	1185	0	47.5
W/C0.45R50	180	400	652	580	580	45.7
W/C0.45R100	180	400	639	0	1138	41.6
W/C0.5R0	180	360	678	1207	0	43.6
W/C0.5R50	180	360	664	591	591	41.2
W/C0.5R100	180	360	651	0	1160	37.4
W/C0.55R0	180	327	689	1226	0	38.2
W/C0.55R50	180	327	674	600	600	35.3
W/C0.55R100	180	327	661	0	1177	34.2

Note: W/C0.45R50 stands for the specimen with W/C of 0.45 and *r*_g_ of 50%.

**Table 6:** Mixture design (kg/m^3^) and compressive strength of steel fiber reinforced recycled coarse aggregate concrete (SFRCAC) with different water content.

Specimen No.	Water	Cement	Sand	NCA	RCA	Steel Fiber	Slump/mm	*f*_cu_/MPa
W16R0	160	400	685	1210	0	0	6	47.8
W16R50	160	400	671	597	597	0	4	45.5
W16R100	160	400	658	0	1171	0	2	45.0
W17R0	170	425	668	1187	0	0	13	51.9
W17R50	170	425	654	582	582	0	7	48.2
W17R100	170	425	642	0	1141	0	5	45.7
W18R0	180	450	650	1157	0	0	32	56.4
W18R50	180	450	636	566	566	0	21	51.3
W18R100	180	450	624	0	1111	0	13	49.2
W19R0	190	475	632	1126	0	0	60	49.6
W19R0MF1.0	190	475	632	1126	0	78	53	48.9
W19R0HF1.0	190	475	632	1126	0	78	42	50.6
W19R0MF2.0	190	475	632	1126	0	156	44	51.0
W19R0HF2.0	190	475	632	1126	0	156	25	52.2
W19R50	190	475	619	551	551	0	46	48.1
W19R50MF1.0	190	475	619	551	551	78	40	49.2
W19R50HF1.0	190	475	619	551	551	78	32	50.5
W19R50MF2.0	190	475	619	551	551	156	34	50.7
W19R50HF2.0	190	475	619	551	551	156	18	50.2
W19R100	190	475	607.3	0	1081	0	29	47.2
W19R100MF1.0	190	475	607	0	1081	78	25	48.6
W19R100HF1.0	190	475	607	0	1081	78	20	49.0
W19R100MF2.0	190	475	607	0	1081	156	22	50.1
W19R100HF2.0	190	475	607	0	1081	156	10	51.3
W20R0	200	500	615	1094	0	0	96	50.2
W20R50	200	500	602	536	536	0	78	48.7
W20R100	200	500	590	0	1051	0	49	51.2
W21R0	210	525	597	1063	0	0	130	47.4
W21R50	210	525	585	521	521	0	121	46.4
W21R100	210	525	574	0/0	1020	0	106	46.0
W22R0	220	550	580	1032	0	0	175	47.2
W22R50	220	550	568	505	505	0	168	44.5
W22R100	220	550	557	0	991	0	159	42.8

Note: W19R50HF1.0 stands for the specimen with water content of 190 (kg/ m^3^), *r*_g_ of 50%, hooked at both ends steel fiber, and *V*_f_ of 1.0%.

**Table 7:** Mixture proportions (kg/m^3^) and test results for new sand content method.

Specimen No.	Water	Cement	Sand	NCA	RCA	Steel Fiber	*β*_s_	Slump/mm	*f*_cu_/MPa
CR0MF0	190	475	622.8	1106	0	0	36	64	50.4
CR50MF0	190	475	648.3	521	521	0	38.4	58	51.6
CR100MF0	190	475	673.2	0	987.4	0	40.8	50	51.2
CR0MF0.5	190	475	629	1085	0	39	36.7	60	50.9
CR50MF0.5	190	475	655.7	511.7	511.7	39	39	57	51.1
CR100MF0.5	190	475	678	0	968.2	39	41.2	48	51.5
CR0MF1.0	190	475	634	1064	0	78	37.3	60	51.2
CR50MF1.0	190	475	660	502	502	78	39.7	53	52.3
CR100MF1.0	190	475	683.8	0	949.6	78	41.9	43	52.6
CR0MF1.5	190	475	641	1044	0	117	38	55	53.3
CR50MF1.5	190	475	667	492	492	117	40.4	50	52.1
CR100MF1.5	190	475	689.1	0	931	117	42.5	40	52.9
CR0MF2.0	190	475	647	1023	0	156	38.7	52	54.5
CR50MF2.0	190	475	670.8	482.2	482.2	156	41	45	53.7
CR100MF2.0	190	475	693.7	0	912.4	156	43.2	38	53.2

Note: CR50MF1.0 stands for the specimen with *r*_g_ of 50%, milling steel fiber and *V*_f_ of 1.0%.

**Table 8:** Mix design details of SFRCAC based on new method, test results of slump and compressive and flexural strength.

Test Value	Specimen No.								
	CR0MF	CR50MF	CR100MF	CR0WF	CR50WF	CR100WF	CR0HF	CR50HF	CR50HF
W/C	0.43	0.41	0.39	0.43	0.41	0.39	0.43	0.41	0.39
Water (kg) /volume%	187.5/ 18.75	190.5/ 19.05	193/ 19.3	187.5/ 18.75	190.5/ 19.05	193/ 19.3	187.5/ 18.75	190.5/ 19.05	193/ 19.3
Cement (kg) /volume%	436/ 14.1	464.6/ 15	494.9/ 15.7	436/ 14.1	464.6/ 15	494.9/ 15.7	436/ 14.1	464.6/ 15	494.9/ 15.7
SFs (kg) /volume%	143.5/ 1.84	143.5/ 1.84	143.5/ 1.84	124.8/ 1.6	124.8/ 1.6	124.8/ 1.6	59.3/ 0.76	59.3/ 0.76	59.3/ 0.76
NCA (kg) /volume%	1054.8/ 37.5	488.4/ 17.3	0/ 0	1064.8/ 37.8	493.7/ 17.5	0/ 0	1100/ 39.1	509/ 19.3	0/ 0
Cement (kg) /volume%	436/ 14.1	464.6/ 15	494.9/ 15.7	436/ 14.1	464.6/ 15	494.9/ 15.7	436/ 14.1	464.6/ 15	494.9/ 15.7
RCA (kg) /volume%	0/ 0	488.4/ 18.5	907.5/ 34.4	0/ 0	493.7/ 18.7	916.5/ 34.7	0/ 0	509/ 18.1	947.7/ 35.9
Sand (kg) /volume%	660.4/ 25.8	674/ 26.4	685.1/ 26.8	656.5/ 25.7	672/ 26.3	681.9/ 26.7	647.1/ 25.3	661.6/ 25.9	672.3/ 26.3
βs/%	38.5	40.8	43	38.1	40.5	42.7	37	39.4	41.5
Slump (mm)	56	60	52	55	53	52	60	62	56
*f*_cu_ (MPa)	50.4	52.6	49.8	51	51.8	49.6	50.6	49.9	49
*f*_ftm_ (MPa)	12.3	12	10.6	11.8	11.3	10.2	10.9	12	12.5

Note: CR50MF stands for the specimen with *r*_g_ of 50% and milling steel fiber.
